# Relationship Between Cell Surface Viral Glycoprotein Expression and Resistance of Parainfluenza Virus Persistently Infected Cells to Complement-Mediated Lysis

**DOI:** 10.3390/pathogens14080815

**Published:** 2025-08-17

**Authors:** Nasser N. Yousef, Griffith D. Parks

**Affiliations:** Burnett School of Biomedical Sciences, College of Medicine, University of Central Florida, Orlando, FL 32827, USA; nasser.yousef@ucf.edu

**Keywords:** paramyxoviruses, persistent infections, complement

## Abstract

Persistent RNA virus infections (PI) are often characterized by extended viral shedding and maintained cycles of inflammation. The innate immune Complement (C′) pathways can recognize acute infected (AI) cells and result in their lysis, but the relative sensitivity of PI cells to C′-directed killing is incompletely understood. Here, we extended our previous studies on the interactions of C′ with parainfluenza virus AI and PI A549 cells to two additional respiratory tract cell lines. AI Hep2 and H1975 cells infected with Parainfluenza virus 5 (PIV5) were found to be highly sensitive to C′ lysis. By contrast, PIV5 PI cells were highly resistant to killing by C″. Surface deposition of membrane attack complex (MAC) and C3 was also greatly reduced on the surface of PI cells compared to AI cells. PI cells had lower levels of surface viral glycoprotein expression compared to AI cells. Treatment of AI cells with ribavirin (RBV) showed a dose-dependent decrease in both viral glycoprotein expression and sensitivity to C′-mediated lysis. When surface viral glycoprotein levels were reduced in AI cells to those in PI cells, AI cells became similarly resistant to C′. While sialic acid levels on PI cell surfaces matched that of naïve cells, enzymatic removal of this sialic acid did not increase sensitivity to C′-mediated lysis. Despite their varying profiles of C′ activation and deposition, these studies indicate downregulation of viral gene expression as a common mechanism of C′ resistance across various parainfluenza virus PI cell lines.

## 1. Introduction

Some RNA viruses establish infections that are persistent (PI), and in which infectious virus is constantly produced from cells at lower levels that an acute infection (AI) [1,2]. These persistent infections (PIs) can be clinically important, due to prolonged damaging inflammation as well as increased viral transmission due to extended viral shedding. PIs can also serve as reservoirs for the emergence of novel viral variants, some of which can have altered infectivity, transmission through populations, and evasion of immune responses [1,3,4,5,6].

A recent example of the complications of prolonged RNA virus infections is seen with SARS-CoV-2 infections, which are strong activators of human inflammation pathways. For example, complement (C′) activation markers in plasma samples from “long COVID” patients are found at elevated levels compared to health individuals [7], and this is proposed to reflect a contribution that C′ makes to prolonged inflammatory damage [8]. In addition, individuals with documented prolonged SARS-CoV-2 infections can show increased frequencies of emergence of distinct viral variants [9]. Thus, there is high significance in uncovering a clear understanding of the molecular and cellular determinants that drive C′ interactions with persistently infected cells.

A number of paramyxoviruses have been shown to establish PIs in human populations, with the most notable example being a PI by measles virus (MeV) [10]. After an initial acute infection, MeV has been shown in some patients to transition to a PI of the nervous system, which can lead to fatal subacute sclerosing panencephalitis (SSPE) [11]. Some paramyxoviruses have also been shown to form persistent infections in bats which can serve as reservoirs for novel viral strains [12,13]. The paramyxoviruses MeV, Mumps virus (MuV), Sendai virus (SeV), and parainfluenza viruses (PIV) can readily establish persistent infections in cell cultures [5,6,14,15,16,17,18,19,20,21]. As a prototypic paramyxovirus, Parainfluenza virus 5 (PIV5) has been previously studied as a model for paramyxoviruses which can readily form PIs in many cell lines [4,14,15]. For PIV5, PI cells can display altered viral polymerase functions [15], IFN signaling [20], and inclusion bodies [4,22]. PIV5 PIs have also been shown to result in the mutants with higher fusogenic capacity [5].

The conversion of an AI to PI for RNA virus infections can change the response of innate immune pathways, thereby allowing escape from host response systems [17,20,23]. As such this dampening of innate responses may help in the survival of PI cells. The innate immune pathways that make up the complement (C′) system can reduce virus burden by neutralization or opsonizing them directly, recruiting leukocytes, and inducing lysis of infected cells [24]. The C′ cascade can be activated through the lectin, classical or alternative pathways. For parainfluenza and related viruses, the most active pathway appears to be the alternative pathway [25]. All three pathways converge on the central protein complement component 3 (C3), which is cleaved upon activation to form the two fragments C3a and C3b. C3b can then form part of the C5 convertase, cleaving C5 into C5a and C5b. C5b joins with C6, C7, and C8 and attaches to the membrane of viruses and infected cells. Several C9 proteins polymerize and bind to C5b-8 forming the membrane attack complex (MAC), a transmembrane pore that ultimately lyses the cell. Given the importance of C′ in anti-viral immunity, the role of C′ in responding to and lysing PI cells remains incompletely understood.

We previously showed that PIV5 activates the alternative C′ pathway and that lysis of PIV5 AI cells by C′ pathways occurs in a C3- and C5-dependent manner [14]. Using A549 lung epithelial cells, our prior work also showed that cells acutely infected with PIV5 were very sensitive to lysis through C′-mediated mechanisms, whereas C′ killing of PIV5 PI cells was negligible [14]. After treatment with C’, PI A549 cells showed elevated levels of C3a and C5a, and high levels of C3 and MAC labeling of the cells surface, but were not found to be killed by C′ treatment [11]. In the current study, we build on our previous findings on the resistance of PIV5 PI lung cells to C′-mediated killing by analyzing two additional respiratory tract cell lines, with the hypothesis that mechanisms of resistance to C′-mediated lysis would be constant across respiratory cells lines. Here, we show a common property that transition of a PIV5 AI of Hep2 and H1975 cells to a PI converts these cells from sensitive to resistant to C′-mediated lysis. However, the properties of these two other respiratory PI cells with respect to C′ activation differ from that seen previously with PI A549 cells. Most importantly, pharmacological reduction of viral protein expression levels in AI cells to those seen in PI cells resulted in AI cells gaining resistance to C′-mediated lysis. Our results highlight the relationship between reduced viral gene expression in a PI and evasion of C′-mediated lysis.

## 2. Materials and Methods

### 2.1. Cell Lines, Viruses, and Treatments

Cultures of H1975 (catalog #CRL-5908; ATCC; Manassas, VA, USA) were maintained in RPMI medium containing 10% fetal calf serum that had been heat inactivated (HI FBS, Gibco, Thermo Fisher Scientific, Waltham, MA, USA). DMEM containing 10% HI FBS was used to maintain growth of HEp2, Vero and CV-1 cells (ATCC; Manassas, VA, USA).

Monolayers of Vero cells were used to amplify virus stocks, as these cells do not produce type I interferon. For titering, dilutions of virus stock were prepared in DMEM, and applied to monolayers of CV-1 cells for one hour (h). After washing, cells were overlayed with DMEM containing 2% agar. After 5 days at 37 °C, monolayers were washed and stained with crystal violet to reveal plaques. The PIV5 mutant containing substitutions in the 3’ leader promoter (referred to as PIV5 here) was used for all of these studies and is expresses GFP as an additional gene as previously described [26]. To generate persistently infected (PI) cell lines, a multiplicity of infection (MOI) of 10 PFU/cell was used to infect H1975 and HEp2 cells with PIV5 and cells were cultured for two weeks. PI cells were then passaged and maintained as done for their naïve counterparts. For cells to be acute infected (AI), virus diluted in RPMI (H1975) or DMEM (HEp2) containing a 1:10 dilution of bovine serum albumin (BSA) and then used for infection for 1 h. Mock infected cells were treated with media alone. Analyses of AI cells were done 24 h post infection (hpi) in all experiments unless otherwise stated. PI cell cultures underwent mock infections and time-zero was defined as the time of media replacement directly following the 1 hr mock-infection incubation.

As a source of C’, normal human serum (NHS) from pooled donors was obtained from Innovative Research (Novi, MI). When indicated, a control serum was generated by for 30 min treatment at 56°.

Ribavirin (RBV, 1-β-d-ribofuranosyl-1,2,4-triazole-3-carboxamide, 196066, MP Biomedicals, Eschwege, Germany) treatments were carried out at various concentrations as detailed in the legend to figures. Briefly, H1975 and HEp2 cells were treated for 24 h (hrs) pre-infection with RBV. Cells were then infected for 1 hr, washed, then cultured in media containing RBV prior to analysis.

Treatments with neuraminidase (NA) from Clostridium perfringens (C. welchi, N5631-5UN, MilliporeSigma, Burlington, MA, USA) were carried out at 1 unit/mL (U/mL).

### 2.2. Cell Viability Assay

H1975 and HEp2 cells were transduced with lentivirus expressing nuclear red fluorescent protein by treatment with IncuCyte Nuclight Red (Sartorius, Göttingen, Germany). Two hundred thousand cells were treated with 1 μL/mL polybrene and then infected with 15,000 transduction units of lentivirus. After 24 h, cells were washed and cultured in DMEM containing 10% HI FBS. After 2 days, transduced cells were then selected for by culturing in DMEM/FBS with 1 μg/mL puromycin.

The IncuCyte SX5 Live-Cell Analysis system (Sartorius) was used to monitor cell killing in real time as described [27,28]. Briefly, 5000 cells/well of H1975-Nuclight Red (NLR) and HEp2-NLR cells were cultured in triplicate, then infected at an MOI of 10 with PIV5, or mock infected. Cultures were maintained for 24 h, and then treated as detailed in individual legends to figures. Plates were maintained inside a 5% CO_2_ incubator within the IncuCyte chamber and microscopic images were captured by the IncuCyte every 2 h using a 10× objective in red, green, and phase channels. Red object count (ROC) corresponding to cellular nuclei are automatically quantified from fluorescent images using Incucyte 2024B software (Sartorius). The values are then normalized to time 0 for all conditions and expressed as a percent through Incucyte 2024B. Calculations for percent cytotoxicity are performed by normalizing these values to the control group as noted in corresponding figure legends. Percent cytotoxicity at each time point was calculated by normalizing NHS-treated samples to heat-inactivated (HI) NHS treated samples as previously detailed [27].

### 2.3. Flow Cytometry

The CytoFLEX Flow Cytometer was used to analyze protein expression (Beckman Coulter, Brea, CA, USA). Software from CytExpert (Beckman Coulter) was used to analyze at least 10,000 events that were independent. After washing in PBS, and incubating for 5 min with trypsin at 37 °C, cells were quenched with DMEM containing 10% HI FBS and pelleted for 3 min at 3000 RPM. Cells were washed in PBS, pelleted as above and then incubated in 100 μL of primary antibody for 30 min on ice. Cells were pelleted, washed in PBS and incubated in 100 μL of secondary antibody in the dark on ice. Cells were then pelleted, washed in PBS, resuspended in PBS and processed for analysis by flow cytometry.

Cell surface PIV5 glycoprotein expression was monitored as described previously [29] by flow cytometry using an anti-PIV5 mouse polyclonal antibody (1:100) followed with staining with AlexaFluor 405 anti-mouse (1:1000, A31553, Thermo Fisher Scientific, Waltham, MA, USA). C3 and MAC surface deposition were monitored using an anti-C3 antibody (1:1000, 204869, Calbiochem, San Diego, CA, USA), or anti-SC5b-9 antibody (1:500, A239, Quidel, San Diego, CA, USA), followed by antibody staining using AlexaFluor405 antibodies specific for the individual species of antibody. Cell surface sialic acid was monitored by flow cytometry using MALL II Maackia Amurensis Lectin II that was biotinylated (1:50, B-1265-1, Vector Laboratories, Newark, CA, USA) and then with treatment with Pacific Blue conjugated Streptavidin (1:1000, S11222, Thermo Fisher Scientific, Waltham, MA, USA). HN levels were monitored using a mouse anti-PIV5 HN 4b [30] monoclonal antibody at 1:1000 followed by treatment with anti-mouse AlexaFluor 405. CD55 surface expression was monitored using an AF405 conjugated anti-human CD55 antibody (R&D Systems, FAB20091V).

### 2.4. RT-qPCR

H1975 and HEp2 cells were cultured in 6-well plates and total RNA was extracted using TRIzol^®^ (Invitrogen, Carlsbad, CA, USA), 1 mL per well), without DNAse treatment. One μg of total RNA was used to generate cDNA using the TaqMan^®^ Reverse Transcription Reagents (Applied Biosystems, Foster City, CA, USA) as described in the manufacturer’s instructions. Quantitative real-time PCR for the fusion (F), hemagglutinin neuraminidase (HN), matrix (M), and nucleocapsid protein (NP) genes was performed using Bio-Rad CFX Connect Real-Time (Bio-Rad, Hercules, CA, USA) and Fast SYBR^®^ FAST Green Master Mix (Applied Biosystems, Foster City, CA, USA). Relative gene expression was calculated using CFX Manager Software version 2.3 ((Bio-Rad). The primers utilized for quantifying corresponding genes are summarized in Table 1. All primers were run using a melting temperature of 60 °C (Table 1).

### 2.5. Statistics

Where statistics are shown, experiments included at least three biological replicates. Statistical analysis was through GraphPad version 10.2.3 one-way ANOVA and two-way ANOVA. The figures indicate *p*-values of: * <0.05, ** <0.01, *** <0.001, **** <0.0001.

## 3. Results

### 3.1. PIV5 PI Cells Have Reduced Viral Gene Expression and Progeny Infectious Virus Production Compared to AI Cells

**Figure 1:**
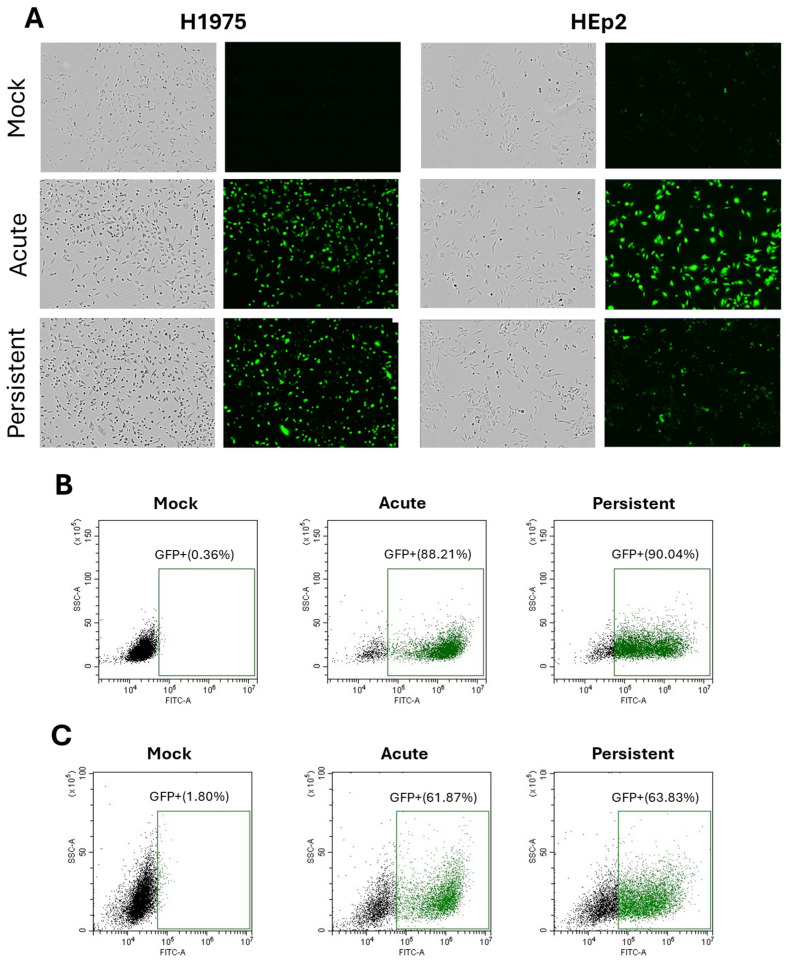
Green fluorescence confirms expression of viral gene products in H1975 and HEp2 AI and PI cells. AI H1975 and HEp2 cells were infected with PIV5 (MOI 10) and processed 24 hpi. Mock-infected cells were also processed 24 hpi and PI cells were treated in parallel with mock infections. (**A**) Phase and green fluorescent images of H1975 and HEp2 mock-infected, AI and PI cells are shown. (**B**,**C**) Mock-infected, AI, and PI H1975 (**B**) and HEp2 (**C**) cells were processed via flow cytometry to quantify green fluorescence.

A PIV5 mutant with substitutions in the leader and encoding GFP (referred to as PIV5 here) was utilized for the following studies due to its potent ability to activate C′ pathways, as described previously [14]. We established persistently infected (PI) cell lines by performing a high MOI acute infection of respiratory tract cell lines H1975 and HEp2, followed by media renewals every 2–3 days for two weeks. As there are no known biomarkers for establishing a parainfluenza virus PI, viable GFP+ cells which survive the AI, produce infectious virus and can be continuously passaged was used as evidence of a PI. Analyses of acute infected (AI) cells were done 24 hpi in all experiments unless otherwise stated. PI cell cultures were mock infected as a control and time-zero was defined as the time of media replacement for all experiments. As shown in the microscopic pictures in Figure 1A, nearly all cells in the PI culture maintained the same morphology as AI cells and were positive for GFP, indicating active expression of viral gene products. When quantified by flow cytometry, PI H1975 cells (Figure 1B) were detected at 90% GFP+ and Hep2 cells were detected as 63% GFP+. For unknown reasons, PI Hep2 cells showed a larger percentage of cells that were scored as GFP- by our gating strategy. This finding of cells with low level viral antigen is similar to that reported previously for PIV5 PI BALB/c mouse embryo cells in vitro (4), and may be a cell type-specific property.

**Figure 2:**
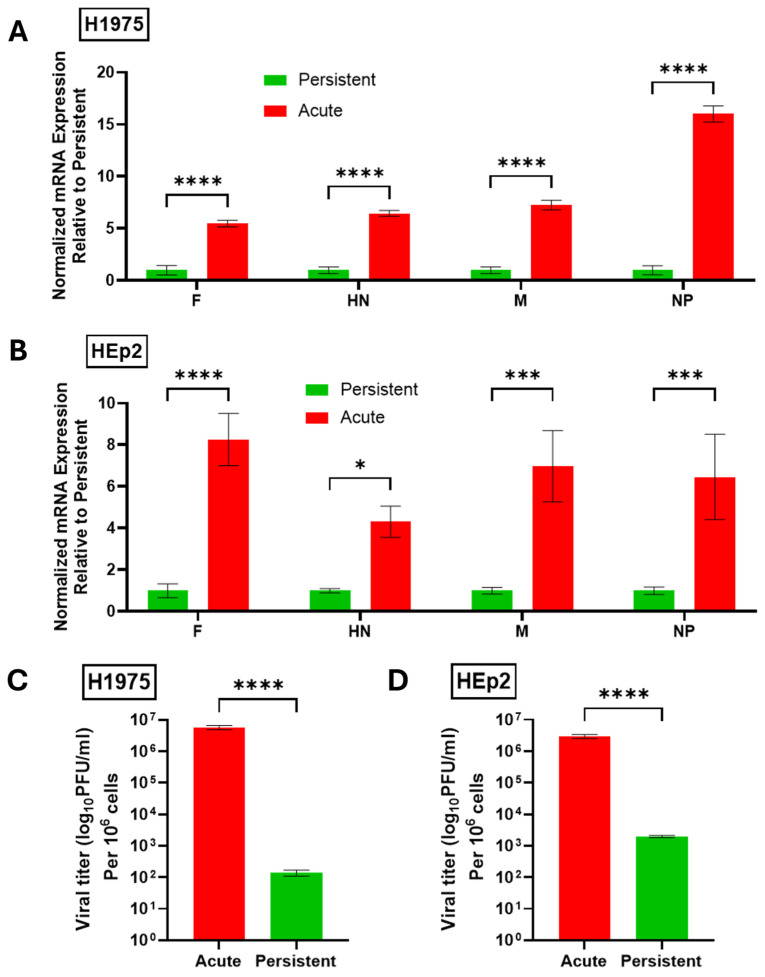
PIV5 PI cells have reduced viral gene expression and progeny infectious virus production compared to AI cells. (**A**,**B**) Naïve or PI H1975 (**A**) or HEp2 (**B**) cells were cultured in 6-well plates. Naïve cells were mock infected as a negative control or acutely infected with PIV5 at an MOI of 10 PFU/cell. Total RNA was collected 24 hpi and analyzed via RT-qPCR for expression of viral genes. Values were normalized to expression of β-actin. Viral gene expression in PI cell cultures was set equal to 1 and expression in AI cell cultures is expressed as fold change relative to PI. (**C**,**D**) Virus released from PIV5 PI H1975 (**C**) or HEp2 (**D**) cells at 24 h after replacing media or from AI cells 24 hpi was collected and quantified via plaque assay. Titers were normalized to 10^6^ cells. *p*-values: * <0.05, *** <0.001, **** <0.0001.

To determine the relative viral gene expression in H1975 and HEp2 PI cells, AI H1975 and HEp2 cells were infected at a high MOI with PIV5 and processed 24 hpi. Mock-infected cells were also processed 24 hpi and PI cells were treated in parallel with mock infections. At 24 hpi, total cellular RNA was collected and the mRNA expression of viral genes was quantified by RT-qPCR. Data were expressed with amount of PI viral RNA set to a value of 1. As shown in Figure 2, both H1975 (Figure 2A) and HEp2 (Figure 2B) AI cells showed a 5–15-fold higher level of expression of all four viral genes when compared to PI cells. For H1975 cells, the largest difference between AI and PI gene expression was seen for the NP gene, while changes in gene expression with Hep2 cells were consistently 4–8 fold lower for PI versus AI. The reduced levels of viral gene products in PI cells versus AI cells are consistent with characteristics of paramyxovirus PI cell cultures described previously [15,31,32].

The relative level of progeny infectious virus produced from AI and PI cultures was quantified. Here, media were collected from AI cells and mock-infected PI cells 24 hpi and titered via plaque assay. As shown in Figure 2C,D, H1975 and HEp2 PI cells showed between ~50,000-fold (H1975) and ~1000-fold (Hep2) reduction in production of infectious progeny virus compared to AI cells. Together these data confirm the successful establishment of H1975 and HEp2 PI cell lines that maintain infectivity and viral gene expression but to a lower level than their AI counterparts.

### 3.2. Differential Sensitivity of PIV5 PI Versus AI Cells to C′-Mediated Lysis

Using H1975 and HEp2 cells which are transduced to express a nuclear red fluorescence protein (NLR), the IncuCyte instrument can record red fluorescent nuclei, reported as red object count (ROC) as a measure of viability in real-time. PI versions of the H1975-NLR and HEp2-NLR cells were generated and gene expression profiles were confirmed as described in Figure 2 above. In the following experiments, naïve cells were treated as a negative control by mock infection or acutely infected

**Figure 3:**
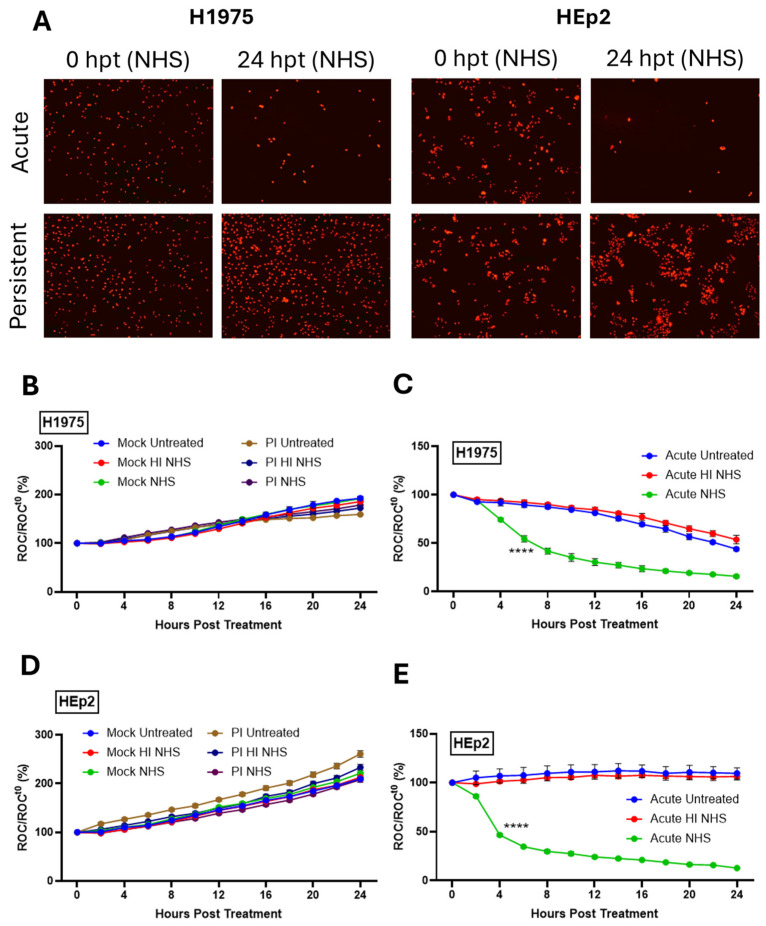
Differential sensitivity of PIV5 PI versus AI cells to C′-mediated lysis. (**A**) Red fluorescent images comparing cell viability of AI and PI H1975-NLR or HEp2-NLR cells were captured 24 hpt with 10% NHS. (**B**–**E**) Naïve and PI H1975-NLR (**B**,**C**) or HEp2-NLR (**D**,**E**) cells were plated at a density of 5000 cells/well. Naïve cells were mock infected as a negative control or acutely infected with PIV5 at an MOI of 10 PFU/cell. Mock-infected, AI, and PI cells were then left untreated, treated with 10% HI NHS, or treated with 10% NHS 24 hpi. Red fluorescent images were captured at 2 hr intervals for 24 h. The IncuCyte recorded Red object count (ROC) per well and these values were normalized to time 0 (ROC^t0^) when sera were added. Values are expressed as a ratio compared to time zero (ROC/ROC^t0^ (%)). **** indicates when a *p*-value of <0.0001 first appears during the time, comparing AI samples with HI NHS versus NHS, and this significance continues throughout all subsequent times.

At 24 hpi, mock-infected naïve cells, AI, or mock-infected PI cells were untreated, or treated with 10% NHS for active C′ factors. As a control, cells were alternatively treated with HI NHS. Cells were then imaged every 2 h using the IncuCyte as described previously [14,29,33]. Figure 3A shows raw red fluorescent images from the IncuCyte comparing AI and PI cells of each cell type at time 0 and 24 hp NHS treatment. For both H1975-NLR and HEp2-NLR cells, NHS treatment resulted in a dramatic loss of the number of cells (as evidenced by fewer red nuclei), whereas PI cells continued to grow and showed no loss of red nuclei.

Real-time kinetics of C′-mediated killing was determined whereby red object count (ROC) at each data point was compared as a ratio to the value at time 0 when sera treatments occurred (ROC/ROC^t0^) and then expressed as a percentage (ROC/ROC^t0^(%)). As shown in Figure 3B,D, mock-infected H1975-NLR and HEp2-NLR cells continue to grow following treatment with NHS. Both H1975-NLR (Figure 3C) and HEp2-NLR (Figure 3E) PIV5 AI cells exhibited sensitivity to C′-mediated lysis after treatment with NHS (green lines), reaching 50% ROC by 6 and 4 h post treatment (hpt), respectively. Most importantly, PI versions of both H1975-NLR (Figure 3B) and HEp2-NLR (Figure 3D) cells showed resistance to C′ lysis (purple lines), with each growing at a similar rate to their mock-infected counterparts (Figure 3B,D). These data demonstrate that PIV5 AI H1975 and HEp2 cells are sensitive to C′-mediated lysis, while their PI counterparts are resistant to lysis.

### 3.3. Surface Deposition of C3 and MAC on PIV5 AI Cells but Not PI Cells

**Figure 4:**
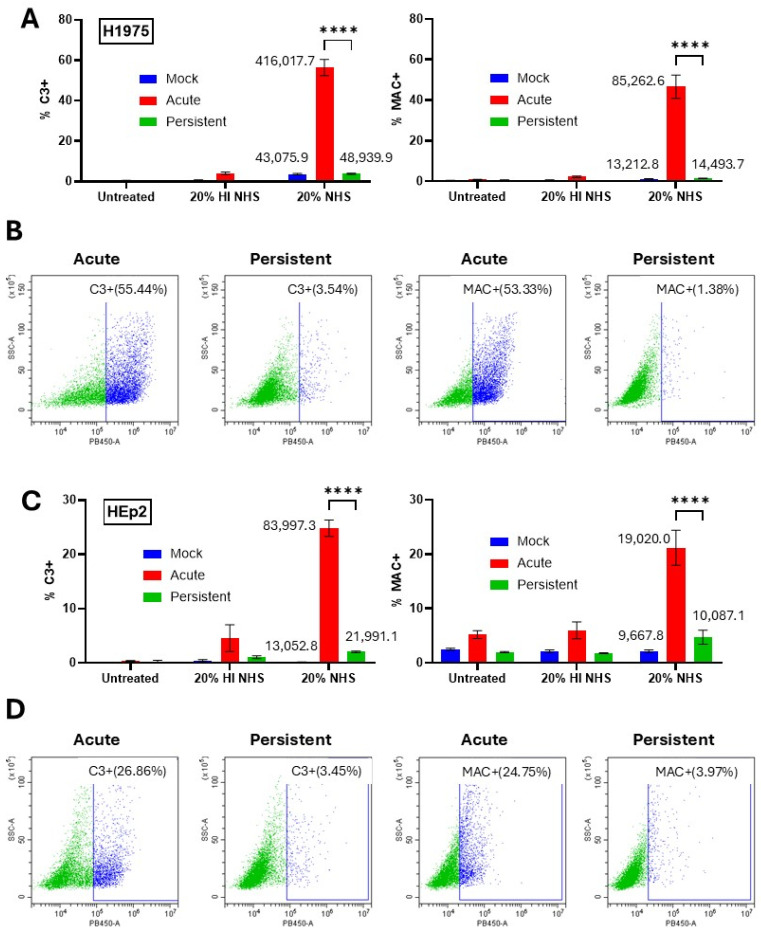
Differential MAC and C3 deposition on PIV5 AI cells but not PI cells. (**A**–**D**) Naïve and PI H1975 (**A**,**B**) and HEp2 (**C**,**D**) cells were cultured in 24-well plates. Naïve cells were mock infected as a negative control or acutely infected with PIV5 at high MOI. At 24 hpi, cells were left untreated or treated with 20% HI NHS as a control or 20% NHS for 30 min. Cells were then stained with antibodies to C3 or C5b-9 before analysis via flow cytometry. Mean fluorescence intensity (MFI) values are shown next to corresponding bars for NHS treated samples. Panels (**B**,**D**) are representative scatter plots of infected cell samples, demonstrating gating strategies. **** indicates *p*-value < 0.0001.

The differential sensitivity of AI versus PI cells to killing by C′ treatment raised the hypothesis that C3 and MAC deposition would also differ between these cell populations. To determine how C3 and MAC deposition compared AI versus PI cells, mock-infected, AI, and PI cells were not treated, treated with 20% HI NHS, or treated with 20% NHS for 30 min. Cells were surface stained with antibodies to either C3 or MAC and analyzed using flow cytometry. Both H1975 (Figure 4A,B) and HEp2 (Figure 4C,D) AI cell populations showed a significant percentage of cells positive for C3 and MAC surface deposition which was not seen with HI NHS treatment. Nearly 55% of H1975 AI cells were positive for C3 and MAC surface deposition. HEp2 AI cells showed greater than 20% of cells positive for both C3 and MAC surface deposition. Most strikingly, PI versions of both H1975 and HEp2 cells both showed minimal positive staining for C3 and MAC surface deposition, matching that seen on mock infected cells. Together, these data show that, unlike their AI cell counterparts, NHS treatment of PI versions of H1975 and HEp2 cells does not lead to appearance of C3 and MAC on the surface.

### 3.4. Manipulating Cell Surface Viral Glycoprotein Levels Alters the Sensitivity to C′-Mediated Killing of PIV5 Infected Cells

**Figure 5:**
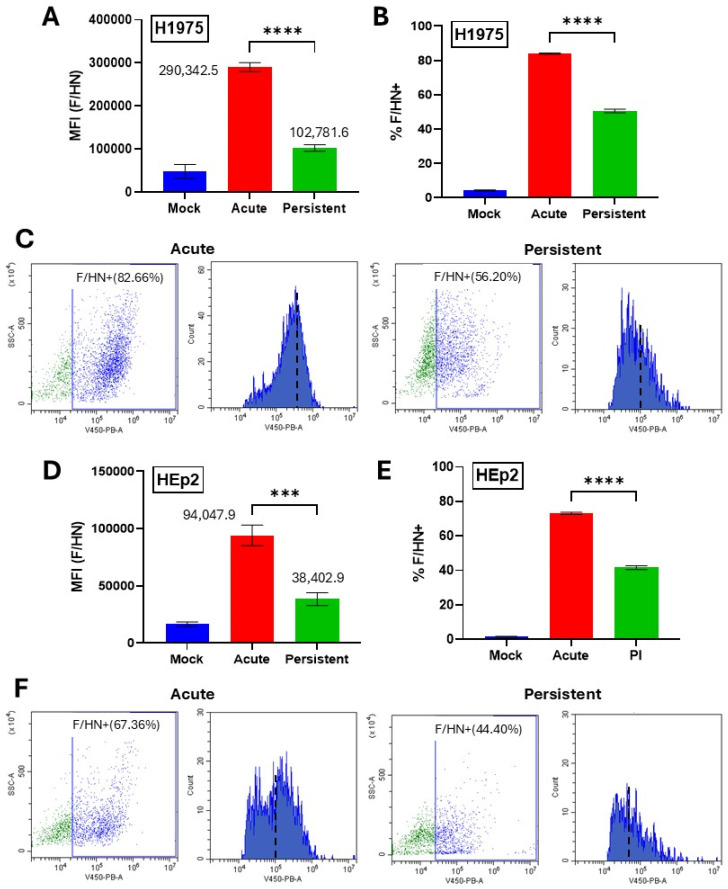
Viral glycoprotein levels are reduced on the surface of PI cells versus AI cells. Naïve and PI H1975 (**A**–**C**) or HEp2 (**D**–**F**) cells were cultured in 24-well plates. Naïve cells were mock infected as a negative control or acutely infected with PIV5 at an MOI of 10 PFU/cell. At 24 hpi, cells were surface stained with a mouse polyclonal anti-PIV5 antibody and analyzed via flow cytometry. (**C**,**F**) Raw flow cytometry scatter plots and histograms are shown of infected cell samples. MFI = Mean Fluorescence Intensity. *** indicates *p*-value < 0.001, **** indicates *p*-value < 0.0001.

Reduced mRNA levels from PI viral genes (Figure 2) and a lack of C3 and MAC deposition on PI cells (Figure 4) raised the hypothesis that the reduced sensitivity to C′ lysis seen with PI cells was due to lower levels of viral proteins on the cell surface which activate the C′ cascade. To determine the relative level of viral glycoproteins on the surface of our PIV5 PI H1975 and HEp2 cells compared to their AI counterparts, mock-infected cells, AI cells, and PI cells were stained using a polyclonal anti-PIV5 antibody then processed using flow cytometry. H1975 (Figure 5A) and HEp2 (Figure 5D) PI cells showed mean fluorescence intensity (MFI) that was reduced to 35% and 41% of their AI counterparts, respectively, indicating lower levels of surface expression of the PIV5 F and/or HN glycoproteins. Here, both viral glycoproteins will be detected, since the serum used in this assay is polyclonal. H1975 cells also showed a decrease in the fraction of cells that were positive for surface glycoprotein expression from ~80% in AI cell cultures to ~50% in PI cell cultures (Figure 5B). Similarly, HEp2 cells showed a reduction from ~70% in AI cell cultures to ~40% in PI cell cultures (Figure 5E). Raw flow cytometry scatter plots and histograms for H1975 (Figure 5C) and HEp2 (Figure 5F) PI versus AI cells show a clear shift in the gated cell populations, indicating lower fluorescence intensity and therefore lower cell surface viral glycoprotein expression in PI populations. Together, these data show that expression of surface viral glycoproteins is significantly reduced on the PI versions of H1975 and HEp2 cells versus their AI counterparts.

**Figure 6:**
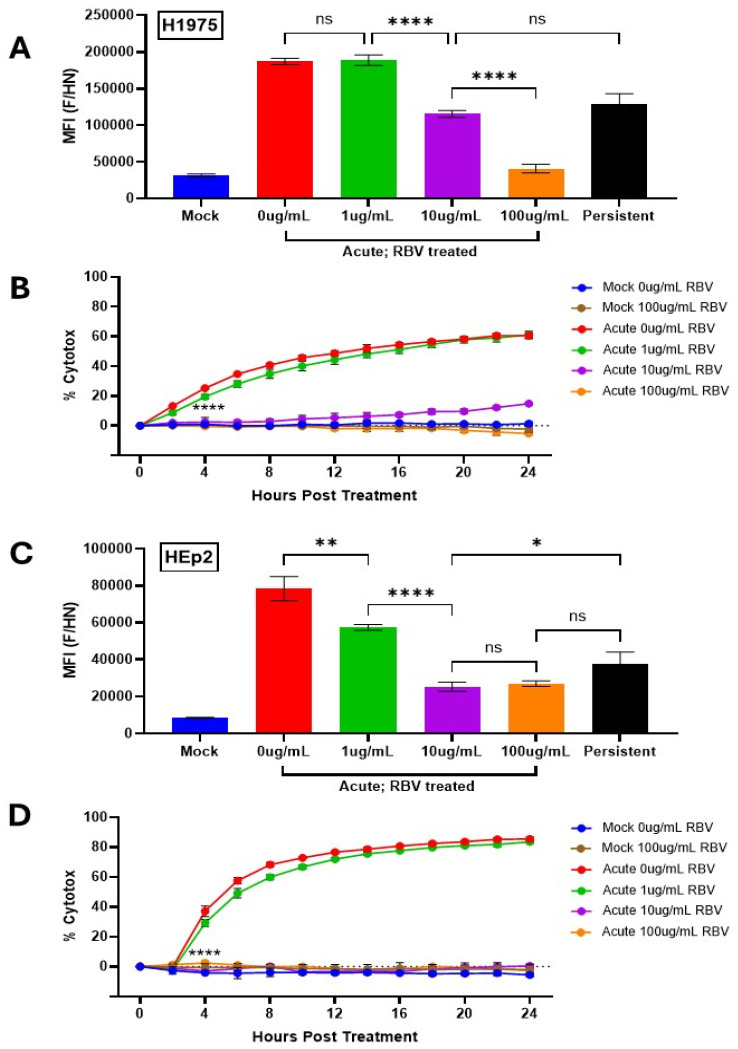
Manipulating cell surface viral glycoprotein levels alters sensitivity to lysis by C′ of PIV5 infected cells. (**A**,**C**) Naïve and PI H1975 (**A**) or HEp2 (**A**) cells were left untreated or treated with RBV at various concentrations (1–100 μg/mL) for 24 h then mock infected or infected with PIV5 at an MOI of 10. Cells were then cultured in media supplemented with various concentrations of RBV (1–100 μg/mL). At 24 hpi, cells were stained with a polyclonal anti-PIV5 antibody and analyzed via flow cytometry. MFI = Mean Fluorescence Intensity. (**B**,**D**) Naïve and PI H1975-NLR (**B**) or HEp2-NLR (**D**) cells were cultured in media alone or media supplemented with various concentrations of RBV (1–100 μg/mL). Naïve cells were mock infected as a negative control or acutely infected with PIV5 at an MOI of 10 PFU/cell, then left untreated or treated with 10% HI NHS or 10% NHS. Red fluorescent images were captured and quantified using the IncuCyte as described in Figure 3. Values of NHS treated wells are expressed as a percentage of HI NHS treated wells and displayed as % cytotoxicity. **** indicates when a *p*-value of <0.0001 is seen in the experiment, comparing untreated versus 10 μg/mL RBV treated AI cells, and this statistical significance remains at continued timepoints. *ns* indicates not significant, * designates *p*-value < 0.05, ** designates *p*-value < 0.01, **** designates *p*-value < 0.0001.

The synthetic nucleoside ribavirin (RBV) has been shown previously to inhibit viral RNA synthesis and gene expression for a large number of RNA viruses, including the paramyxovirus HPIV2 which is closely related to PIV5 [34]. While the exact mechanism is not entirely clear, available data indicates that RBV reduces viral gene expression by inhibition of cellular inosine monophosphate dehydrogenase and incorporation of abnormal nucleotides into viral RNA which results in lethal mutations [35]. To manipulate the levels of surface PIV5 proteins, H1975 and HEp2 cells were pre-treated for 24 h with RBV in increasing doses before being mock infected or infected with PIV5 at a high MOI. Immediately following infection, the cells were cultured in RBV supplemented media. At 24 hpi, cells were stained with anti-PIV5 antibodies and analyzed by flow cytometry. As shown in Figure 6A, PIV5 AI H1975 cells showed a dose-dependent reduction in MFI with increasing doses of RBV, indicating a gradual reduction in surface expression of viral glycoproteins. It is noteworthy that treatment with 10 μg/mL RBV (purple bar, Figure 6A) resulted in an MFI closely matching that seen for H1975 PI cells (black bar, Figure 6A). Similar results were seen with HEp2 AI cells (Figure 6C) treated with RBV at 10 μg/mL, where the MFI of viral glycoprotein surface expression was similar to the PI counterpart (compare purple to black bars, Figure 6C).

To determine how increasing doses of RBV altered sensitivity of AI cells to C′-mediated lysis, naïve and PI H1975-NLR and HEp2-NLR cells were incubated with increasing levels of RBV and then infected with PIV5 at high MOI as outlined above. At 24 hpi, cells were left untreated, treated with 10% NHS that was HI, or treated with 10% NHS. Samples were then placed in the IncuCyte for real-time imaging and analysis as described for Figure 3. Samples treated with 10% NHS were normalized to their control samples treated with 10% HI NHS at each time point and values are expressed as percentage cytotoxicity (% cytotox) to better display cell death caused specifically by active C′ proteins.

H1975-NLR (Figure 6B) and HEp2-NLR (Figure 6D) AI cells left untreated or treated with only 1 μg/mL RBV retained sensitivity to C′-medicate cytotoxicity (red and green lines). Most importantly, at 10 μg/mL RBV treatment, when the MFI of AI cells was found to be similar to that of PI cells, H1975-NLR AI cells show a dramatic reduction in percentage cytotoxicity from nearly 60% in untreated AI cells to ~15% by 24 hpt (Figure 6B, compare purple and red lines). Similarly, HEp2-NLR AI cells showed a striking reduction from ~80% to 0% cytotoxicity with 100 μg/mL RBV treatment (Figure 6D). It is noteworthy that while H1975 AI cells treated with 10 μg/mL RBV had similar MFI for PIV5 glycoproteins as PI cells (Figure 6A), they still had slightly higher cytotoxicity compared to PI cells (see 24 h timepoint of purple line Figure 6B). This indicates that additional factors other than surface glycoprotein levels may factor into resistance to killing by C’.

### 3.5. Cell Surface Sialic Acid Levels on PI Cells Are Similar to Naïve Cells

**Figure 7:**
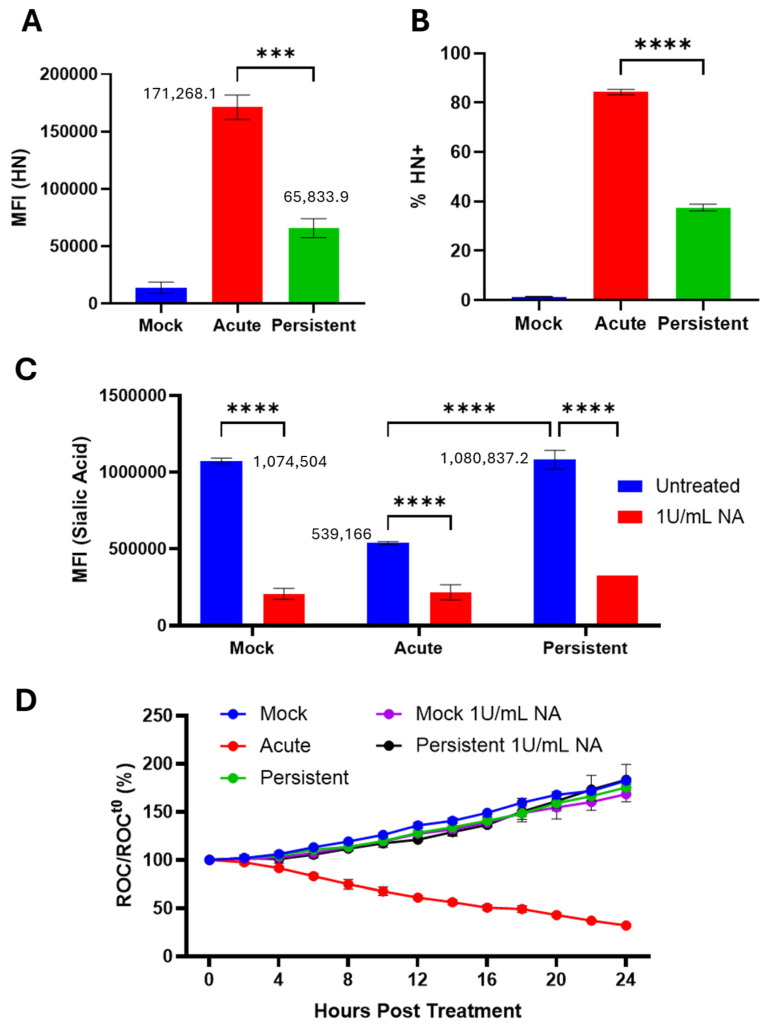
Cell surface sialic acid levels on PI cells are similar to naïve cells. (**A**,**B**) Naïve and PI H1975 cells were plated in a 24-well plate. Naïve cells were mock infected as a negative control or acutely infected with PIV5 at an MOI of 10 PFU/cell. The cells were then stained with an anti-PIV5 HN monoclonal antibody and processed via flow cytometry. (**C**) Mock-infected, AI, and PI cells were treated with 1 U/mL *Clostridium perfringens* neuraminidase (NA) immediately following infection. At 24 hpi, cells were stained with the lectin MAL II followed by conjugated streptavidin, then analyzed via flow cytometry. MFI = mean fluorescence intensity. (**D**) Mock-infected, AI and PI cells were treated with NA immediately following infection. At 24 hpi, cells were left untreated, treated with 10% HI NHS, or treated with 10% NHS. Red fluorescent images were captured and quantified using the IncuCyte as described in Figure 3. *** and **** show *p*-values of <0.001 and <0.0001.

The PIV5 HN protein exhibits neuraminidase activity, cleaving various sialic acid linkage types including α2–3-linked sialic acid [36]. To test this hypothesis, mock-infected, AI, and PI H1975 cells were stained using an anti-PIV5 HN monoclonal antibody and processed via flow cytometry for cell surface HN levels. As shown in Figure 7A, there was a 2.6-fold reduction of HN expression on the surface of H1975 PI cells versus AI cells. The percentage of cells that express surface HN protein was similarly reduced from ~80% in AI cell populations to ~40% in PI cell populations (Figure 7B).

To determine if this reduction in HN expression correlated with higher cell surface sialic acid, mock-infected, AI, and PI cells were left untreated or treated immediately following infection with *Clostridium perfringens* NA which cleaves α2,3-, α2,6-, and α-2,8-linked sialic acid. Cells were then incubated with biotinylated MAL II, a lectin that binds α2–3-linked sialic acids [37] and analyzed as described in the legend to Figure 7. As shown in Figure 7C, H1975 AI cells showed a 2-fold lower level of lectin staining compared to mock infected cells, consistent with the catalytic activity of HN. These levels were reduced further by NA treatment. Strikingly, a comparison of MFI levels for PI cells versus mock infected cells showed ~99.4% identity, and both were higher than that seen with AI cells. These levels were also lower after NA treatment.

To test if removal of cell surface sialic acid on PI cells was sufficient to increase their sensitivity to C′-mediated lysis [38], mock-infected, AI, and PI H1975-NLR cells were treated with NA according to the procedure outlined for Figure 7C. Cells were then left untreated, treated with 10% HI NHS as a control, or treated with 10% NHS, then imaged using the IncuCyte following the procedures outlined in Figure 3 above. AI cells were sensitive to C′-mediated lysis as expected (red line Figure 7D), whereas mock-infected and PI cells showed no significant difference in sensitivity to C′-mediated lysis after treatment with NA (Figure 7D).

### 3.6. Expression of C′ Regulators on H1975 and HEp2 PI Cells Does Not Correlate with Sensitivity to C′-Mediated Lysis

**Figure 8:**
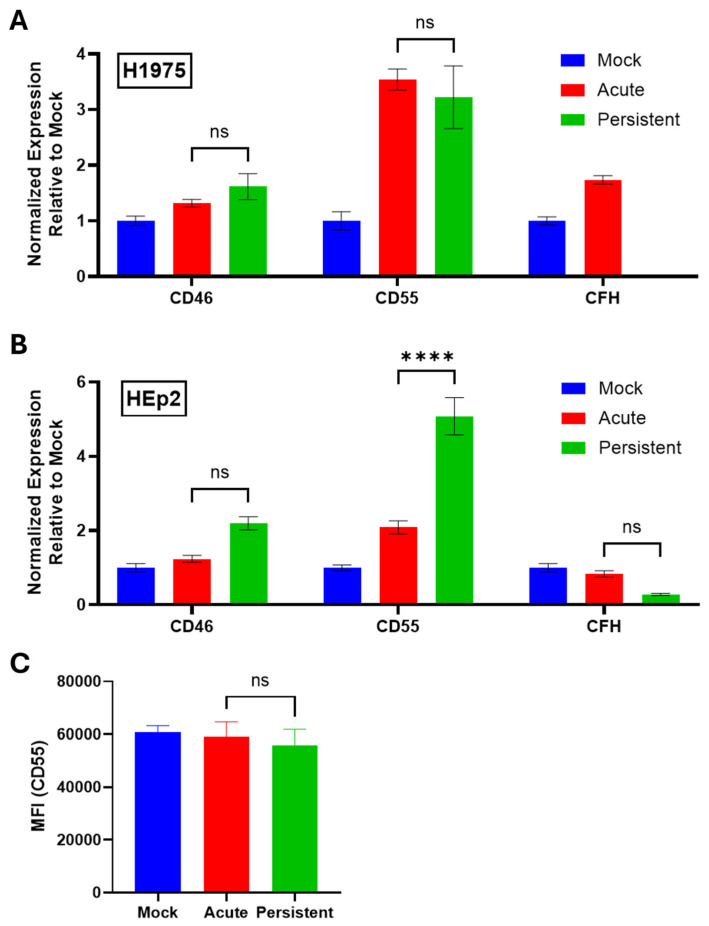
C′ regulators are not significantly upregulated in H1975 and HEp2 PI cells. (**A**,**B**) RNA samples from H1975 (**A**) and HEp2 (**B**) cells were collected as described in the legend to Figure 2. Samples were analyzed via RT-qPCR to determine gene expression of three C′ regulators: CD46, CD55, and CFH. Values were normalized to expression of β-actin. C′ inhibitor expression in mock-infected cell cultures was set equal to 1 and expression in AI and PI cel cultures is expressed as fold change relative to mock. (**C**) Mock-infected, AI, and PI HEp2 cells were stained with an anti-CD55 antibody and processed via flow cytometry. MFI; Mean Fluorescence Intensity. ns stands for not significant, **** indicates *p*-value < 0.0001.

To determine how the expression of cell intrinsic C′ regulators is altered in H1975 and HEp2 PI cells, RNA was collected according to the procedures outlined in Figure 2A,B and analyzed via RT-qPCR for expression of three early-stage C′ regulators: CD46, CD55, and CFH. Strikingly, H1975 and HEp2 PI cells each showed unique C′ regulator expression profiles. H1975 PI cells showed no significant upregulation of CD46, CD55, or CFH (Figure 8A) when compared to AI cells, while HEp2 PI cells showed a 2.5-fold upregulation of CD55 mRNA expression (Figure 8B) relative to their AI cell counterparts.

To determine if cell surface CD55 protein expression on HEp2 PI cells was similarly increased, mock-infected, AI, and PI HEp2 cells were stained with an anti-CD55 antibody and processed via flow cytometry. HEp2 PI cells showed no significant upregulation of CD55 surface expression compared to mock-infected and AI cells (Figure 8C). Thus, changes in CD55 mRNA did not correlate with changes in cell surface expression of CD55 protein.

## 4. Discussion

Persistent infections are a major global human health problem, driving prolonged inflammatory responses, increasing viral transmission, and serving as reservoirs for novel variants. A recent and remarkable example of this is the prevalence of long COVID in some patients following acute SARS-CoV-2 infection. It has been hypothesized that in some cases of long COVID, SARS-CoV-2 infection lingers but with reduced expression and virion production, resulting in chronic low-level C′ activation [39]. In the case of the positive strand RNA virus Chikungunya Virus, infected individuals show elevated levels of C′ fragments which correlated with stages of virus-induced acute to chronic disease [40]. Likewise, Hepatitis C virus inhibits C′ responses at multiple levels, including synthesis of C′ factors, activity of C′ pathways and induction of C′ inhibitors [23], all of which are thought to contribute to persistent infections of liver cells and prolonged inflammatory responses. Thus, PI cells could maintain low level C′ activation while evading C′-mediated lysis, resulting in cycles of damaging inflammatory responses.

Given our previous studies on the evasion of A549 PI lung cells from C′ [14], we extended our work to additional PI respiratory tract cells with the goal of determining common and differential molecular mechanisms of resistance to C′-mediated lysis. Our results support a hypothesis on a common mechanism of resistance across three respiratory cell lines based on PI cells having a decreased level of surface glycoproteins needed to activate the C′ pathways to the final cell lysis stage.

Consistent with the typical phenotype associated with persistent viral infections [14,15,21,31,32], our PIV5 PI laryngeal (HEp2) and lung epithelial (H1975) cell lines showed lower viral gene and protein expression as well as reduced production of progeny infectious virus when compared to their AI counterparts. As shown previously, this is likely in part due to the differential phosphorylation of the viral P protein that occurs during transition from an AI to a PI. This has been proposed to disrupt interactions between P and L (two subunits of the RDRP) and therefore reduce viral polymerase activity [15]. Remarkably, NHS-treated PIV5 PI HEp2 and H1975 respiratory cell lines were resistant to C′-mediated lysis and showed minimal levels of C3 and MAC deposition on their surface, matching that of mock-infected control cells. This finding contrasts with the previous finding of high deposition of these C′ proteins on the surface of PI A549 cells [14]. The presence of C3 and MAC deposition on PI A549 cells [14], combined with the lack of C′-driven lysis, suggested active late-stage inhibition of C′ as opposed to failure to activate C′ pathways. By contrast, our results here showing the lack of C3 and MAC deposition entirely on the HEp2 and H1975 PI cells suggested either a very early-stage inhibition of C′ or a lack of C′ activation altogether.

CD46, CFH, and CD55 each work as early-stage regulators of the C′ cascade. CD46, also referred to as membrane cofactor protein (MCP), is a transmembrane C′ regulator that serves as a cofactor for the C′ Factor I (CFI) protease. CD46 can bind C3b on the cell surface, allowing CFI to cleave C3b into inactive fragments. This directly prevents C′ activation and amplification by reducing the C3b available for formation of C3 and C5 convertases [41,42]. The MeV hemagglutinin (H) glycoprotein has been shown to bind to CD46 for entry into cells and this interaction downregulates CD46 expression on the cell surface, increasing sensitivity to C′-mediated lysis [43,44,45]. CFH is a soluble C′ regulator that similarly acts as a cofactor to CFI for C3b cleavage and has decay-accelerating activity against the C3 convertase by competing with C′ factor B (CFB) for binding to C3b [46,47]. CD55 can also accelerate the dissociation of the C3 convertase. Each of these C′ regulators therefore prevent C3b binding on the surface of cells. Upon exploring the gene expression of these three C′ regulators, PI H1975 cells showed no significant upregulation while PI HEp2 cells showed ~2.5-fold upregulation in mRNA expression of CD55 compared to AI cells (Figure 8B). However, changes in CD55 protein cell surface expression did not correlate with mRNA expression, indicating that it is unlikely that CD55 is playing a role in the lack of susceptibility of HEp2 PI cells to C′-mediated killing.

Our most striking finding was that the viral polymerase inhibitor ribavirin could be used to manipulate levels of surface viral protein expression in our AI cells to the same levels seen in our PI cell lines, and that this resulted in the AI cells gaining resistance to C′-mediated lysis. This correlation is consistent with our recent findings that reduction of surface PIV5 glycoprotein expression by co-infection of PIV5 with defective viral genomes (DVGs) similarly showed a dose-dependent decrease in sensitivity to C′-mediated lysis [29]. This manipulation of cell surface viral protein expression by RBV and DI genomes supports a direct correlation between levels of cell surface viral protein expression and sensitivity to C′ lysis in AI cell cultures. Most importantly, when viral protein levels in AI cells are reduced to those seen in PI cells, these AI cells become similarly resistant to C′-mediated lysis. Together with our finding of low deposition of C3 on PI HEp2 and H1975 cell lines, our data lead to a new hypothesis that PI cells down regulate glycoprotein expression to levels that are too low to trigger activation of C3 cleavage and deposition.

Cell surface viral proteins can serve as an “altered self” signal to activate C′ through various pathways. For example, the HIV gp160 protein by itself can activate the classical C′ arm [48], while the MeV F glycoprotein has been shown to activate the alternative C′ pathway [49]. This suggests that direct interactions between F protein and C′ proteins may be a trigger for C′ activation, and reduced F protein reduces activation of C3 cleavage. The higher density of PIV5 glycoproteins on the surface of an AI cell may also create an optimal platform for C′ component deposition, and the lower glycoprotein density on PI cell surfaces may be inadequate for this optimal platform to form. The specific interactions between PIV5 glycoproteins and C′ components remain to be explored and are an important area of research.

The HN viral protein could also be a trigger for C′ activation, through structural motifs or enzymatic activity. Prior work revealed that the level of sialic acid on enveloped viruses correlates with their ability to activate the alternative C′ pathway [38]. Previous studies have also demonstrated that CFH binds α2–3-linked sialic acid, mediating the prevention of C′ activation on the surface of healthy cells [50,51,52]. The HN-mediated removal of sialic acid on the surface of cells, therefore, removes a binding site for CFH and can potentially contribute to increased C′ activation at the cell surface. We hypothesized that reduced HN expression in PIV5 PI cells would result in increased sialic acid surface expression, contributing to evasion of C’. Remarkably, lectin staining for levels of sialic acid levels on PI cells was at 94% of that found on naïve cells, raising the question of whether the restoration of sialic acid levels in PI cells is simply due to reduced HN expression below a threshold or if the neuraminidase activity of PI cell HN is somehow compromised by inhibitors or mutations. Nonetheless, the important result was that reduction of lectin staining (reflecting reduced sialic acid) was not sufficient to confer sensitivity to C′ mediated lysis. This could indicate alternative binding sites for CFH, or that the expression of other surface C′ regulators alone is adequate to prevent initiation of the C′ cascade. Further, these data indicated that it is not the catalytic activity of HN that correlates PIV5 glycoprotein surface expression levels with C′-mediated lysis. Rather, it is likely that C′ detection and activation is due to direct interactions of C′ proteins with viral glycoproteins on the cell surface.

Our findings provide evidence for a mechanism for how RNA virus PIs can avoid inactivation by C′ innate immune pathways. While modulation of C′ responses as a treatment to prevent the damage caused by long-term immune activation is currently in clinical applications [53], future work will be needed to understand the consequences of suppressing C′ pathways on persistent respiratory viral infections.

## Figures and Tables

**Table 1:** Primers utilized for RT-qPCR).

Gene	Primer (Forward)	Primer (Reverse)
β-Actin	5′ GATCATTCGTCCTCCTGAGC 3′	5′ ACTCCTGCTTGCTGATCCAC 3′
F	5′ ACGTGTTATGGTGACTGGCA 3′	5′ GAACAGCACGAATCGAGTGA 3′
HN	5′ TGACCAACCCTTCGTCTACC 3′	5′ CTTGACCGCTTGATCCAAAT 3′
M	5′ TCATGAGCCACTGGTGACAT 3′	5′ TGGAATTCCCTCAGTTGTCC 3′
NP	5′ TGACCAGTCACCAGAAGCTG 3′	5′ CGGAATCAACGAAAGGTGTT 3′
CD46	5′ TGGCTACCTGTCTCAGATGACG 3′	5′ GCATCTGATAACCAAACTCGTAAG 3′
CD55	5′ TGACCCGTTGCCAGAGTGCAG 3′	5′ TGATGAAGGAGAGTGGAGTGGC 3′
CFH	5′ GTCTCCTGACCTCCCAATATG 3′	5′ TCCACCACTTCACTGTGT 3′

## Data Availability

The raw data supporting the conclusions of this article will be made available by the authors on request. GEO data referred to in the text is available through NCBI accession PRJNA1089547PRJNA1089547.

## References

[1] Randall R.E., Griffin D.E. (2017). Within host RNA virus persistence: Mechanisms and consequences. Curr. Opin. Virol..

[2] Randall RE R.W., Kingsbury D.W. (1991). Paramyxovirus persistence. Consequences for host and virus. The Paramyxoviruses.

[3] Marques A.D., Graham-Wooten J., Fitzgerald A.S., Sobel Leonard A., Cook E.J., Everett J.K., Rodino K.G., Moncla L.H., Kelly B.J., Collman R.G. (2024). SARS-CoV-2 evolution during prolonged infection in immunocompromised patients. mBio.

[4] Fearns R., Young D.F., Randall R.E. (1994). Evidence that the paramyxovirus simian virus 5 can establish quiescent infections by remaining inactive in cytoplasmic inclusion bodies. J. Gen. Virol..

[5] Young D.F., Didcock L., Randall R.E. (1997). Isolation of highly fusogenic variants of simian virus 5 from persistently infected cells that produce and respond to interferon. J. Virol..

[6] Rima B.K., Duprex W.P. (2005). Molecular mechanisms of measles virus persistence. Virus Res..

[7] Baillie K., Davies H.E., Keat S.B.K., Ladell K., Miners K.L., Jones S.A., Mellou E., Toonen E.J.M., Price D.A., Morgan B.P. (2024). Complement dysregulation is a prevalent and therapeutically amenable feature of long COVID. Med.

[8] Cervia-Hasler C., Bruningk S.C., Hoch T., Fan B., Muzio G., Thompson R.C., Ceglarek L., Meledin R., Westermann P., Emmenegger M. (2024). Persistent complement dysregulation with signs of thromboinflammation in active Long Covid. Science.

[9] Sigal A., Neher R.A., Lessells R.J. (2025). The consequences of SARS-CoV-2 within-host persistence. Nat. Rev. Microbiol..

[10] Rima B.K. (1999). Paramyxoviruses and chronic human diseases. Bone.

[11] Garg R.K., Mahadevan A., Malhotra H.S., Rizvi I., Kumar N., Uniyal R. (2019). Subacute sclerosing panencephalitis. Rev. Med. Virol..

[12] Gibson L., Ribas M.P., Kemp J., Restif O., Suu-Ire R.D., Wood J.L.N., Cunningham A.A. (2021). Persistence of Multiple Paramyxoviruses in a Closed Captive Colony of Fruit Bats (*Eidolon helvum*). Viruses.

[13] Johnson R.I., Tachedjian M., Clayton B.A., Layton R., Bergfeld J., Wang L.F., Marsh G.A. (2019). Characterization of Teviot virus, an Australian bat-borne paramyxovirus. J. Gen. Virol..

[14] Fox C.R., Yousef N.N., Varudkar N., Shiffer E.M., Aquino J.R., Kedarinath K., Parks G.D. (2025). Resistance to complement-mediated lysis of parainfluenza virus 5-infected cells is acquired after transition from acute to persistent infection. J. Virol..

[15] Young D.F., Wignall-Fleming E.B., Busse D.C., Pickin M.J., Hankinson J., Randall E.M., Tavendale A., Davison A.J., Lamont D., Tregoning J.S. (2019). The switch between acute and persistent paramyxovirus infection caused by single amino acid substitutions in the RNA polymerase P subunit. PLoS Pathog..

[16] Kurebayashi Y., Bajimaya S., Watanabe M., Lim N., Lutz M., Dunagan M., Takimoto T. (2021). Human parainfluenza virus type 1 regulates cholesterol biosynthesis and establishes quiescent infection in human airway cells. PLoS Pathog..

[17] Fujii N., Kimura K., Murakami T., Indoh T., Ishida S., Fujinaga K., Oguma K. (1990). Suppression of interferon-induced oligo-2′,5′-adenylate synthetase induction in persistent infection. J. Gen. Virol..

[18] Genoyer E., Lopez C.B. (2019). The Impact of Defective Viruses on Infection and Immunity. Annu. Rev. Virol..

[19] Manzoni T.B., Lopez C.B. (2018). Defective (interfering) viral genomes re-explored: Impact on antiviral immunity and virus persistence. Future Virol..

[20] Young D.F., Chatziandreou N., He B., Goodbourn S., Lamb R.A., Randall R.E. (2001). Single amino acid substitution in the V protein of simian virus 5 differentiates its ability to block interferon signaling in human and murine cells. J. Virol..

[21] Fox C.R., Parks G.D. (2018). Parainfluenza Virus Infection Sensitizes Cancer Cells to DNA-Damaging Agents: Implications for Oncolytic Virus Therapy. J. Virol..

[22] Carlos T.S., Young D.F., Schneider M., Simas J.P., Randall R.E. (2009). Parainfluenza virus 5 genomes are located in viral cytoplasmic bodies whilst the virus dismantles the interferon-induced antiviral state of cells. J. Gen. Virol..

[23] Patra T., Ray R.B., Ray R. (2019). Strategies to Circumvent Host Innate Immune Response by Hepatitis C Virus. Cells.

[24] Gasque P. (2004). Complement: A unique innate immune sensor for danger signals. Mol. Immunol..

[25] Hirsch R.L., Wolinsky J.S., Winkelstein J.A. (1986). Activation of the alternative complement pathway by mumps infected cells: Relationship to viral neuraminidase activity. Arch. Virol..

[26] Manuse M.J., Parks G.D. (2009). Role for the paramyxovirus genomic promoter in limiting host cell antiviral responses and cell killing. J. Virol..

[27] Varudkar N., Oyer J.L., Copik A., Parks G.D. (2021). Oncolytic parainfluenza virus combines with NK cells to mediate killing of infected and non-infected lung cancer cells within 3D spheroids: Role of type I and type III interferon signaling. J. Immunother. Cancer.

[28] Fox C.R., Parks G.D. (2021). Complement Inhibitors Vitronectin and Clusterin Are Recruited from Human Serum to the Surface of Coronavirus OC43-Infected Lung Cells through Antibody-Dependent Mechanisms. Viruses.

[29] Aquino J.R., Fox C.R., Parks G.D. (2025). Role of Defective Interfering Particles in Complement-Mediated Lysis of Parainfluenza Virus-Infected Cells. Viruses.

[30] Randall R.E., Young D.F., Goswami K.K., Russell W.C. (1987). Isolation and characterization of monoclonal antibodies to simian virus 5 and their use in revealing antigenic differences between human, canine and simian isolates. J. Gen. Virol..

[31] Homann H.E., Hofschneider P.H., Neubert W.J. (1990). Sendai virus gene expression in lytically and persistently infected cells. Virology.

[32] Doi T., Kwon H.J., Honda T., Sato H., Yoneda M., Kai C. (2016). Measles virus induces persistent infection by autoregulation of viral replication. Sci. Rep..

[33] Shiffer E.M., Oyer J.L., Copik A.J., Parks G.D. (2024). Parainfluenza Virus 5 V Protein Blocks Interferon Gamma-Mediated Upregulation of NK Cell Inhibitory Ligands and Improves NK Cell Killing of Neuroblastoma Cells. Viruses.

[34] Kihira S., Uematsu J., Kawano M., Itoh A., Ookohchi A., Satoh S., Maeda Y., Sakai K., Yamamoto H., Tsurudome M. (2014). Ribavirin inhibits human parainfluenza virus type 2 replication in vitro. Microbiol. Immunol..

[35] Leyssen P., Balzarini J., De Clercq E., Neyts J. (2005). The predominant mechanism by which ribavirin exerts its antiviral activity in vitro against flaviviruses and paramyxoviruses is mediated by inhibition of IMP dehydrogenase. J. Virol..

[36] Welch B.D., Yuan P., Bose S., Kors C.A., Lamb R.A., Jardetzky T.S. (2013). Structure of the parainfluenza virus 5 (PIV5) hemagglutinin-neuraminidase (HN) ectodomain. PLoS Pathog..

[37] Dugan A.S., Gasparovic M.L., Atwood W.J. (2008). Direct correlation between sialic acid binding and infection of cells by two human polyomaviruses (JC virus and BK virus). J. Virol..

[38] McSharry J.J., Pickering R.J., Caliguiri L.A. (1981). Activation of the alternative complement pathway by enveloped viruses containing limited amounts of sialic acid. Virology.

[39] Zelek W.M., Harrison R.A. (2023). Complement and COVID-19: Three years on, what we know, what we don’t know, and what we ought to know. Immunobiology.

[40] Kasbe R., Tripathy A.S., Wani M.R., Mullick J. (2024). Elevated Complement Activation Fragments and C1q-Binding Circulating Immune Complexes in Varied Phases of Chikungunya Virus Infection. Curr. Microbiol..

[41] Persson B.D., Schmitz N.B., Santiago C., Zocher G., Larvie M., Scheu U., Casasnovas J.M., Stehle T. (2010). Structure of the extracellular portion of CD46 provides insights into its interactions with complement proteins and pathogens. PLoS Pathog..

[42] Janeway C.A., Travers P., Walport M., Shlomchik M.J. (2001). Immunobiology: The Immune System in Health and Disease.

[43] Schnorr J.J., Dunster L.M., Nanan R., Schneider-Schaulies J., Schneider-Schaulies S., ter Meulen V. (1995). Measles virus-induced down-regulation of CD46 is associated with enhanced sensitivity to complement-mediated lysis of infected cells. Eur. J. Immunol..

[44] Delpeut S., Noyce R.S., Siu R.W., Richardson C.D. (2012). Host factors and measles virus replication. Curr. Opin. Virol..

[45] Lin L.T., Richardson C.D. (2016). The Host Cell Receptors for Measles Virus and Their Interaction with the Viral Hemagglutinin (H) Protein. Viruses.

[46] Fraczek L.A., Martin B.K. (2010). Transcriptional control of genes for soluble complement cascade regulatory proteins. Mol. Immunol..

[47] Hourcade D.E., Mitchell L.M., Medof M.E. (1999). Decay acceleration of the complement alternative pathway C3 convertase. Immunopharmacology.

[48] Thieblemont N., Haeffner-Cavaillon N., Weiss L., Maillet F., Kazatchkine M.D. (1993). Complement activation by gp160 glycoprotein of HIV-1. AIDS Res. Hum. Retrovir..

[49] Devaux P., Christiansen D., Plumet S., Gerlier D. (2004). Cell surface activation of the alternative complement pathway by the fusion protein of measles virus. J. Gen. Virol..

[50] Schmidt C.Q., Hipgrave Ederveen A.L., Harder M.J., Wuhrer M., Stehle T., Blaum B.S. (2018). Biophysical analysis of sialic acid recognition by the complement regulator Factor H. Glycobiology.

[51] Blaum B.S., Hannan J.P., Herbert A.P., Kavanagh D., Uhrin D., Stehle T. (2015). Structural basis for sialic acid-mediated self-recognition by complement factor H. Nat. Chem. Biol..

[52] Ram S., Sharma A.K., Simpson S.D., Gulati S., McQuillen D.P., Pangburn M.K., Rice P.A. (1998). A novel sialic acid binding site on factor H mediates serum resistance of sialylated Neisseria gonorrhoeae. J. Exp. Med..

[53] Azoulay E., Zuber J., Bousfiha A.A., Long Y., Tan Y., Luo S., Essafti M., Annane D. (2024). Complement system activation: Bridging physiology, pathophysiology, and therapy. Intensive Care Med..

